# Analysis of the risk of oncological adverse events associated with infliximab in combination with azathioprine compared to monotherapy: insights from the FAERS database

**DOI:** 10.3389/fphar.2024.1507196

**Published:** 2025-01-08

**Authors:** Qian Qiao, Jiachen Sun, Ya Zheng, Yingying Mi, Yanan Gong, Jiahui Liu, Wenyue Rui, Yumei Ma, Yongning Zhou, Min Liu

**Affiliations:** ^1^ The First Clinical Medical College, Lanzhou University, Lanzhou, China; ^2^ Department of Gastroenterology, The First Hospital of Lanzhou University, Lanzhou, China; ^3^ Gansu Province Clinical Research Centre for Digestive Diseases, The First Hospital of Lanzhou University, Lanzhou, China

**Keywords:** infliximab, azathioprine, combined treatment, tumor, adverse event, FAERS

## Abstract

**Objective:**

This study aimed to evaluate the risk of tumor formation with infliximab or azathioprine monotherapy versus their combination, using the FDA Adverse Event Reporting System (FAERS) database.

**Methods:**

Data were extracted from the FAERS database for patients treated with infliximab, azathioprine, and combination therapy from Q1 2004 to Q2 2024. Signal mining employed methods such as Reported Odds Ratio (ROR), Proportional Reporting Ratio (PRR), Multiple Gamma-Poisson Scaling Assessment (MGPSA) and Bayesian Confidence Interval Progressive Neural Network (BCPNN).

**Results:**

Our analysis of the FAERS database revealed that the highest number of reported cases involved skin-related tumors, both individually and in combination. In terms of sex, the risk of cancer was higher in men compared to women in the infliximab-only and combination groups; however, no sex difference was observed in the azathioprine-only group. Regarding age, we noted an increasing incidence of adverse tumor events in middle-aged and elderly individuals compared to minors, except in the azathioprine group, where age was not identified as an independent risk factor. Additionally, body weight was not found to be an independent risk factor in any of the three medication groups. After controlling for age, sex, and body weight, combination therapy did not increase the risk of tumor development compared to the azathioprine group alone. In contrast, for patients using infliximab alone, combination therapy not only did not elevate the risk of tumor development but also appeared to reduce it. The results of the Weber distribution suggest a random failure-type profile for the infliximab and azathioprine-only group, while an early failure-type profile was observed for the combination therapy. Furthermore, we analyzed the median time to onset and cumulative incidence rates, revealing no significant differences in median time to tumor onset or cumulative incidence rates between the combination therapy and the single agent.

**Conclusion:**

After adjusting for age, sex, and body weight, combination therapy did not significantly increase tumor development risk compared to the azathioprine-only group. Additionally, in patients on infliximab monotherapy, combination therapy appeared to reduce the risk of tumor development.

## 1 Introduction

Autoimmune diseases represent a category of disorders characterized by the immune system’s loss of tolerance to self-antigens, resulting in tissue damage and inflammatory responses that can adversely affect target organs. Recent epidemiological studies have indicated an increasing prevalence of various autoimmune diseases, which carries significant implications for public health (Miller, 2023). Conventional therapeutic agents, such as glucocorticoids and immunosuppressants, are effective in the management of autoimmune diseases; however, they frequently pose challenges in halting disease progression and may result in adverse effects. As research progresses, it has become increasingly clear that cytokines play a crucial role in the dysregulation of autoimmune diseases. Therefore, the introduction of biologic therapies signifies a significant advancement in the treatment of these conditions (Peyrin-Biroulet et al., 2021). The inhibition of TNF-α has been shown to significantly enhance patient functionality and overall quality of life (Ranjan et al., 2022). The efficacy of biologic therapies is, however, constrained, as evidenced by a high rate of loss of response. In the context of inflammatory bowel disease, approximately 40 percent of patients demonstrate a response to biologics and maintain clinical remission after 1 year of treatment (Syversen et al., 2022).

National and international experts consistently provide clear recommendations regarding the utilization of anti-TNF-α agents (Robinson and Keating, 2005; Bauerfeind et al., 2006; Hemperly and Vande Casteele, 2018). Infliximab, a chimeric monoclonal antibody targeting tumor necrosis factor-alpha (TNF-α), exhibits significant anti-inflammatory and immunosuppressive effects through its binding to TNF-α, thereby inhibiting its interaction with tumor necrosis factor receptors (TNFR). This mechanism leads to a decrease in inflammatory responses and a mitigation of disease symptoms. Notably, infliximab was the first TNF-α inhibitor to receive approval from the U.S. Food and Drug Administration (FDA) (Hemperly and Vande Casteele, 2018). Azathioprine is considered a first-line immunosuppressant in the treatment of autoimmune diseases (Atreya and Neurath, 2008). Both pharmaceuticals are now extensively utilized in the management of autoimmune disorders, particularly inflammatory bowel disease. Evidence indicates that the combination of infliximab and azathioprine offers several benefits, including enhanced response rates and elevated remission rates (Bots et al., 2018; Colombel et al., 2019; Panaccione et al., 2014; Sultan et al., 2017), a reduction in inflammatory responses, and the facilitation of mucosal healing (Panaccione et al., 2014),Additionally, this combination has been associated with a decreased incidence of infusion reactions,among other advantages (O’Meara et al., 2014). Additionally, it presents a higher incremental cost-effectiveness ratio compared to infliximab alone (Saito et al., 2013),while also demonstrating improvements in quality-adjusted life years (Ruffolo et al., 2010; Scott et al., 2015). These findings offer clinicians significant therapeutic alternatives that may enhance patients’ symptoms and overall quality of life, while also presenting more effective strategies for the management of autoimmune diseases. However, several studies have indicated that the administration of infliximab in conjunction with azathioprine may elevate the risk of tumorigenesis when compared to the use of a monotherapy (Matsuoka et al., 2018; Lichtenstein et al., 2014). Further investigation is necessary to evaluate the impact of infliximab in conjunction with azathioprine treatment concerning the risk of tumorigenesis.

The FDA Adverse Event Reporting System (FAERS) is a publicly accessible database managed by the U.S. Food and Drug Administration. It facilitates the collection of reports concerning adverse drug events from a wide range of individuals, including healthcare professionals, consumers, and manufacturers. The primary objective of this database is to enhance the FDA post-market safety surveillance of pharmaceuticals and biologics. Utilizing the FAERS data for adverse event database mining is an effective method for identifying potential associations between medications and adverse events. The comprehensive and frequently updated FAERS database facilitates more precise analysis of adverse reaction data, thereby providing a more accurate representation of real-world research trends. In recent years, extensive spontaneous adverse drug reaction (ADR) reporting system databases have become increasingly essential in pharmacovigilance studies for the assessment of drug safety (Morris et al., 2024; Zhou et al., 2023). There have been no data mining studies utilizing the FAERS database that assess the risk of tumorigenesis in patients treated with the combination of infliximab and azathioprine (Li et al., 2024; Ma et al., 2024). This study utilized the FAERS database to examine the disparities in the risk of tumorigenesis among patients receiving infliximab in conjunction with azathioprine compared to those treated with either infliximab or azathioprine alone. Furthermore, the analysis included an evaluation of the time-to-onset characteristics and the differences among the various treatment groups. The findings of this study offer significant insights into the application and management of clinical medications, thereby enhancing the understanding of the impact of these medications on the risk of tumorigenesis.

## 2 Methods

### 2.1 Data sources and preprocessing

The data utilized in this study were sourced from the FAERS database, which encompasses all reports of ADRs collected from the inception of the database up to the second quarter of 2024. This data was subsequently imported into the R programming language for the purpose of collation. The dataset comprises several components, including Patient Demographic and Management Information (DEMO), Drug and Biological information (DRUG), Patient Outcome (OUTC), Adverse Event (REAC), report sources (RPSR), start and end dates of drug therapy (THER), and indications for use/diagnosis (INDI).

**FIGURE 1:**
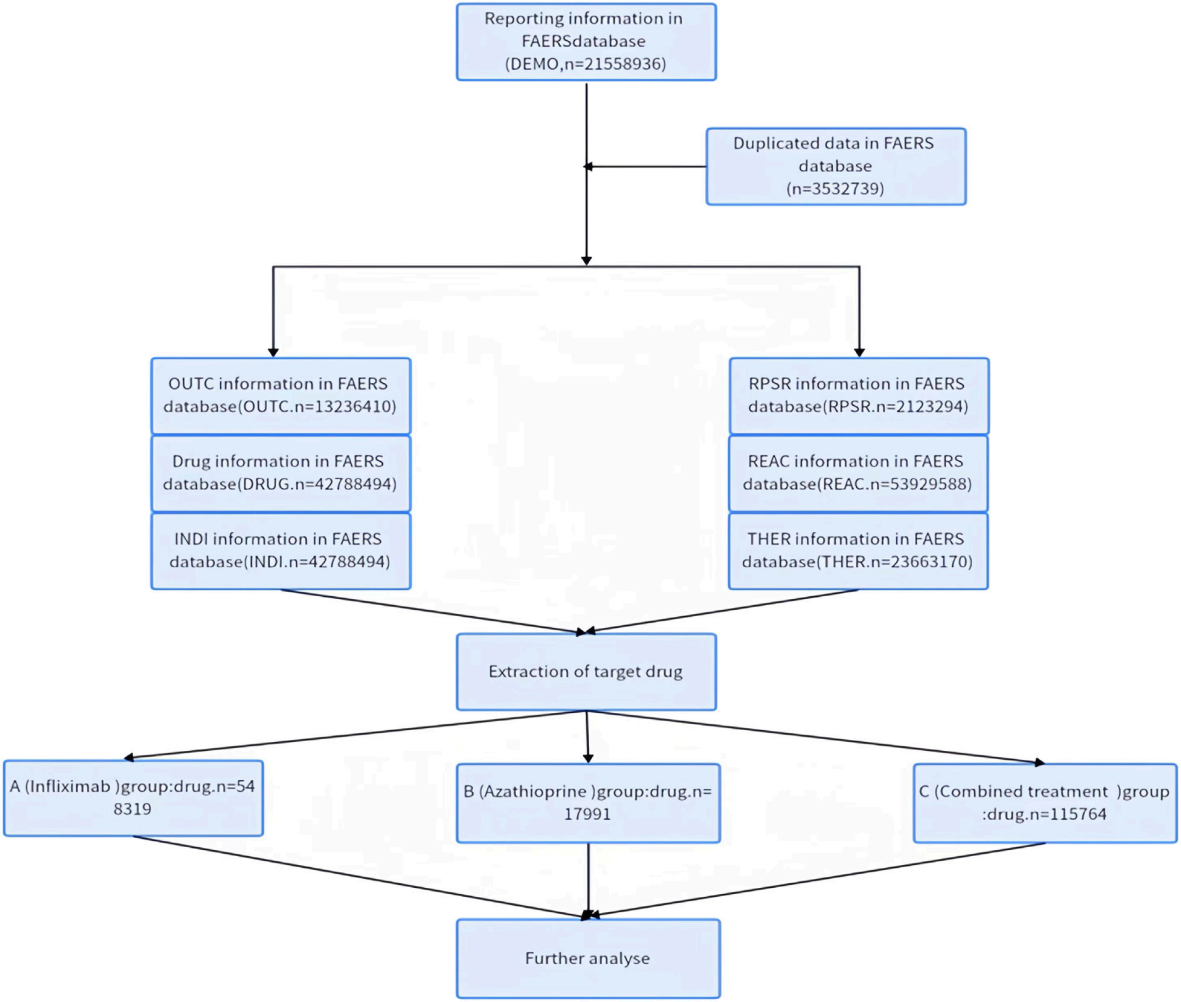
Flowchart illustrating the screening process for adverse reactions based on inclusion criteria.

The collected raw data were filtered to remove duplicates using the DEMO method, as detailed in Figure 1. Drug data inclusion criteria are specified as follows: 1. For the Drug A (Infliximab) group: inclusion criteria encompass suspected drugs containing Drug A (Primary Suspect Drug, PS), with Drug B excluded from the drug combination (Secondary Suspect Drug, SS; Concomitant, C; Interacting, I). 2. For the Drug B (Azathioprine) group: inclusion criteria require suspected drugs containing Drug B (PS), with the drug combination excluding Drug A (SS, C, I). 3. For the Drug A+ Drug B group: inclusion criteria cover suspected drugs containing both Drug A (PS) + Drug B (SS, C, I) or Drug B (PS) + Drug A (SS, C, I). Three sets of adverse reaction reports were obtained by utilizing the abbreviations for infliximab and azathioprine, along with subject-specific and free-text keywords sourced from the PubMed and Embase databases. These reports were subsequently screened for those specifically related to tumor-associated adverse reactions. The included Adverse Drug Event (ADE) reports were classified and described utilizing the System Organ Class (SOC) and Preferred Term (PT) as defined in the Medical Dictionary for Regulatory Activities (MedDRA) adverse drug reaction terminology. In this context, SOC refers to the classification of ADEs, while PT denotes the standardized nomenclature for ADEs.

### 2.2 Statistical analysis

Given the inherent limitations of spontaneous reporting systems in calculating the incidence of adverse events (AEs), this study employed a multifaceted approach to statistically analyze the data. The objective was to minimize the occurrence of false-positive and false-negative signals while ensuring adequate sensitivity. The methodology integrates several joint detection techniques, specifically the Reported Odds Ratio (ROR), Proportional Reporting Ratio (PRR), Multiple Gamma-Poisson Scaling Assessment (MGPSA) and Bayesian Confidence Interval Progressive Neural Network (BCPNN). An AE is classified as a significant signal when it concurrently satisfies the criteria established by all four algorithms. The mathematical formulas and corresponding criteria for these algorithms are presented in Supplementary Table S3. In addition, potential risk factors within each group were evaluated through univariate and multivariate logistic regression analyses, and comparisons were conducted between single and combination drug treatments (Yang et al., 2024). Analyses of the time to onset were conducted by subtracting the date of treatment initiation from the date of the event. Only reports containing time to onset data were included in the analysis. The distribution of time to onset was evaluated using the Weibull shape parameter (WSP) test, and the time to onset was assessed through the Wilcoxon rank sum test (Abe et al., 2016; Nakamura et al., 2015; Sauzet et al., 2013) and cumulative incidence rates were compared across the various groups using the log-rank test (Kinoshita et al., 2020). Statistical analyses and graphical representations were conducted utilizing R Studio (version 4.3.2), IBM SPSS Statistics 27, and Microsoft Excel. The application of these methodologies and tools ensured the accuracy and reliability of the data, thereby establishing a robust foundation for the study’s findings.

## 3 Results

### 3.1 Clinical baseline characteristics

Table 1 provides a comprehensive overview of the clinical characteristics of the patients. Regarding sex distribution, females constituted the majority in both the groups receiving infliximab or azathioprine as monotherapy, as well as in the cohort receiving the combination therapy of infliximab and azathioprine. In relation to body weight, the absence of data was more pronounced; however, the highest number of patients recorded fell within the 50–100 kg range. The patient population was predominantly aged between 18 and 45 years. Regarding treatment indications, both the combination therapy group and the infliximab-only group were extensively utilized for digestive disorders, accounting for 84.93% and 53.58% of cases, respectively. In contrast, the azathioprine-only group exhibited a broader spectrum of indications, with only 28.82% of patients receiving this treatment for digestive disorders. In the context of reporting, physicians comprised the predominant group of reporters, whereas lawyers represented the category with the least frequency of reports. Concerning outcomes, “other” outcomes were the most frequently observed, followed by “hospitalization”, while “permanent impairment” was identified as the least common outcome recorded. Supplementary Figure S1 illustrates the number of reported cases across different years for infliximab alone, azathioprine, and their combination. In the infliximab-only and combination groups, the highest number of reported cases recorded in 2023 was 12,660 and 1,138, respectively. Conversely, the azathioprine-only group experienced its peak incidence of reported cases in 2019, with a total of 533 cases.

**TABLE 1:** Clinical characteristics in different groups.

Characteristics	Infliximab (n, %)	Azathioprine (n, %)	Combined treatment (n, %)
Sex			
Female	76,146 (51.83)	2,472 (46.80)	5,763 (48.53)
Male	53,191 (36.21)	1951 (36.94)	4,762 (40.10)
Missing	17,567 (11.96)	859 (16.26)	1,349 (11.36)
Weight			
<50 kg	8,611 (5.86)	126 (1.93)	928 (7.82)
50∼100 kg	53,850 (36.66)	544 (10.3)	4,980 (41.94)
>100 kg	8,175 (5.56)	102 (1.93)	666 (5.61)
Missing	76,268 (51.92)	4,510 (85.38)	5,300 (44.64)
Age			
<18	12,502 (8.51)	293 (5.55)	1,212 (10.21)
18–45	32,348 (22.02)	1,340 (25.37)	3,911 (32.94)
45–60	22,551 (15.35)	982 (18.59)	1,903 (16.03)
60–75	18,627 (12.68)	805 (15.24)	1,128 (9.5)
>75	4,810 (3.27)	239 (4.52)	164 (1.38)
Missing	56,066 (38.17)	1,623 (30.73)	3,556 (29.95)
Indications			
Gastrointestinal disorders	77,930 (53.58)	1,412 (28.82)	10,021 (84.93)
others	67,510 (46.42)	3,488 (71.18)	1,778 (15.07)
Reporter			
MD	42,193 (28.72)	1,269 (24.02)	4,018 (33.84)
CN	36,285 (24.7)	733 (13.88)	2028 (17.08)
OT	35,193 (23.96)	1,256 (23.78)	3,386 (28.52)
PH	32,105 (21.85)	1,489 (28.19)	2,369 (19.95)
LW	91 (0.06)	5 (0.09)	10 (0.08)
Missing	1,037 (0.71)	530 (10.03)	63 (0.53)
Outcome			
OT	92,296 (60.57)	2,984 (51.34)	8,157 (56.91)
HO	43,054 (28.26)	1,787 (30.75)	4,478 (31.24)
DE	6,355 (4.17)	498 (8.57)	581 (4.05)
LT	4,615 (3.03)	259 (4.46)	529 (3.69)
DS	2,088 (1.37)	66 (1.14)	229 (1.6)
CA	350 (0.23)	83 (1.43)	58 (0.4)
RI	262 (0.17)	60 (1.03)	9 (0.06)
Missing	3,354 (2.2)	75 (1.29)	291 (2.03)

Notes: Reporter: MD: physician; CN: consumer; OT: other health professional; PH: pharmacist; LW: lawyer. Outcome: OT: other; HO: hospital; DE: death; LT: life threaten; DS: disability; CA: congenital abnormality; RI: permanent impairment.

### 3.2 Statistical analysis of adverse events in different treatment groups

**FIGURE 2:**
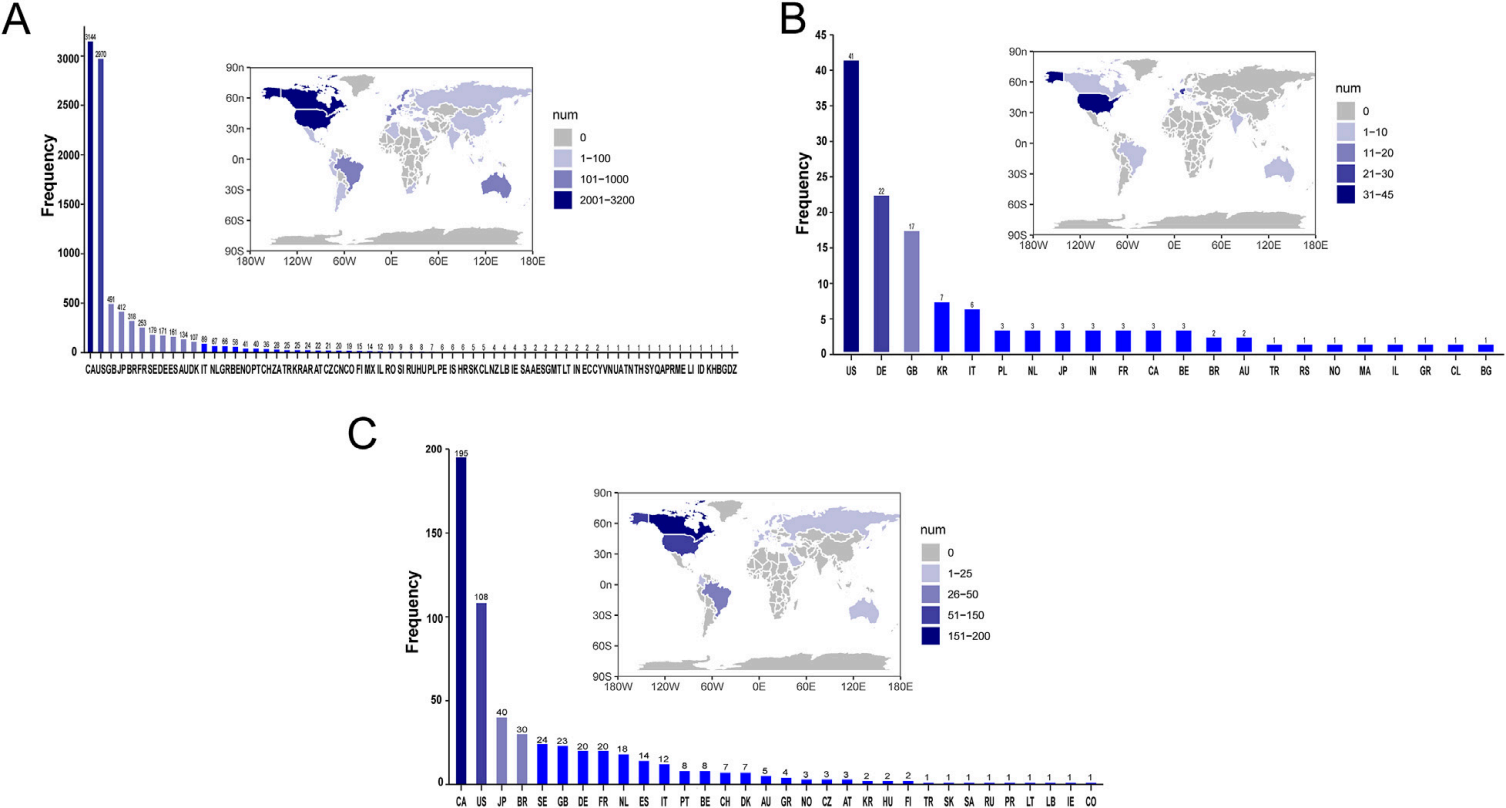
Number of Reported Cases of Tumor-Related Adverse Events by Treatment Group in Each Country. **(A)** The number of reported cases of tumor-related adverse events in the infliximab group for each country. **(B)** The number of reported cases of tumor-related adverse events in the azathioprine group for each country. **(C)** The number of reported cases of tumor-related adverse events in the combined treatment group for each country.

**FIGURE 3:**
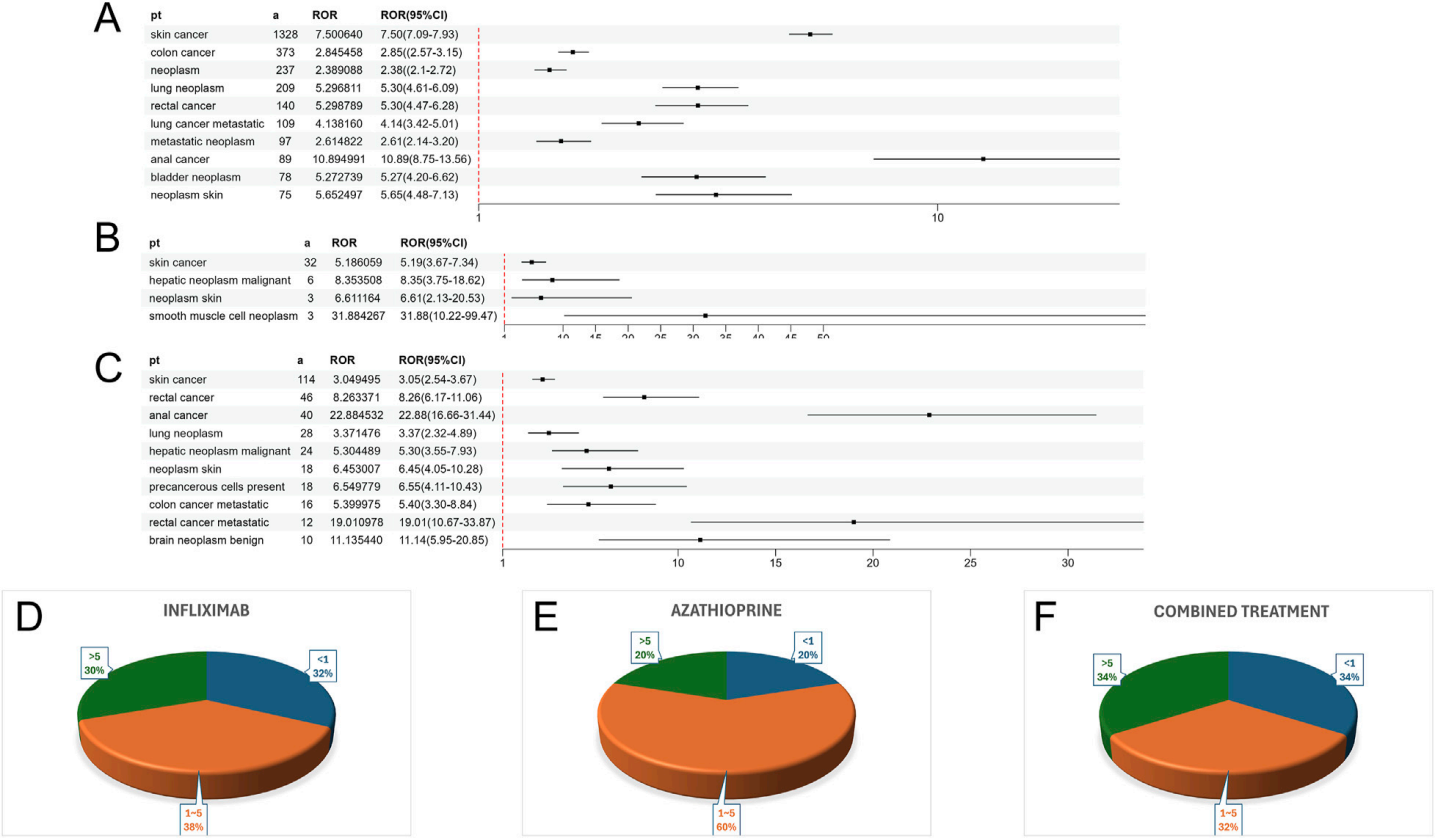
**(A)** ROR values and their 95% confidence intervals for the ten most frequently observed tumor-associated significant signals in the infliximab group. **(B)** ROR values and their 95% confidence intervals for all observed tumor-associated significant signals in the azathioprine group. **(C)** ROR values and their 95% confidence intervals for the ten most frequently observed tumor-associated significant signals in the combined treatment group. **(D)** Distribution of Time to Confirmed Tumor Positivity in the Infliximab Group. **(E)** Distribution of Time to Confirmed Tumor Positivity in the azathioprine Group. **(F)** Distribution of Time to Confirmed Tumor Positivity in the combined treatment Group.

**FIGURE 4:**
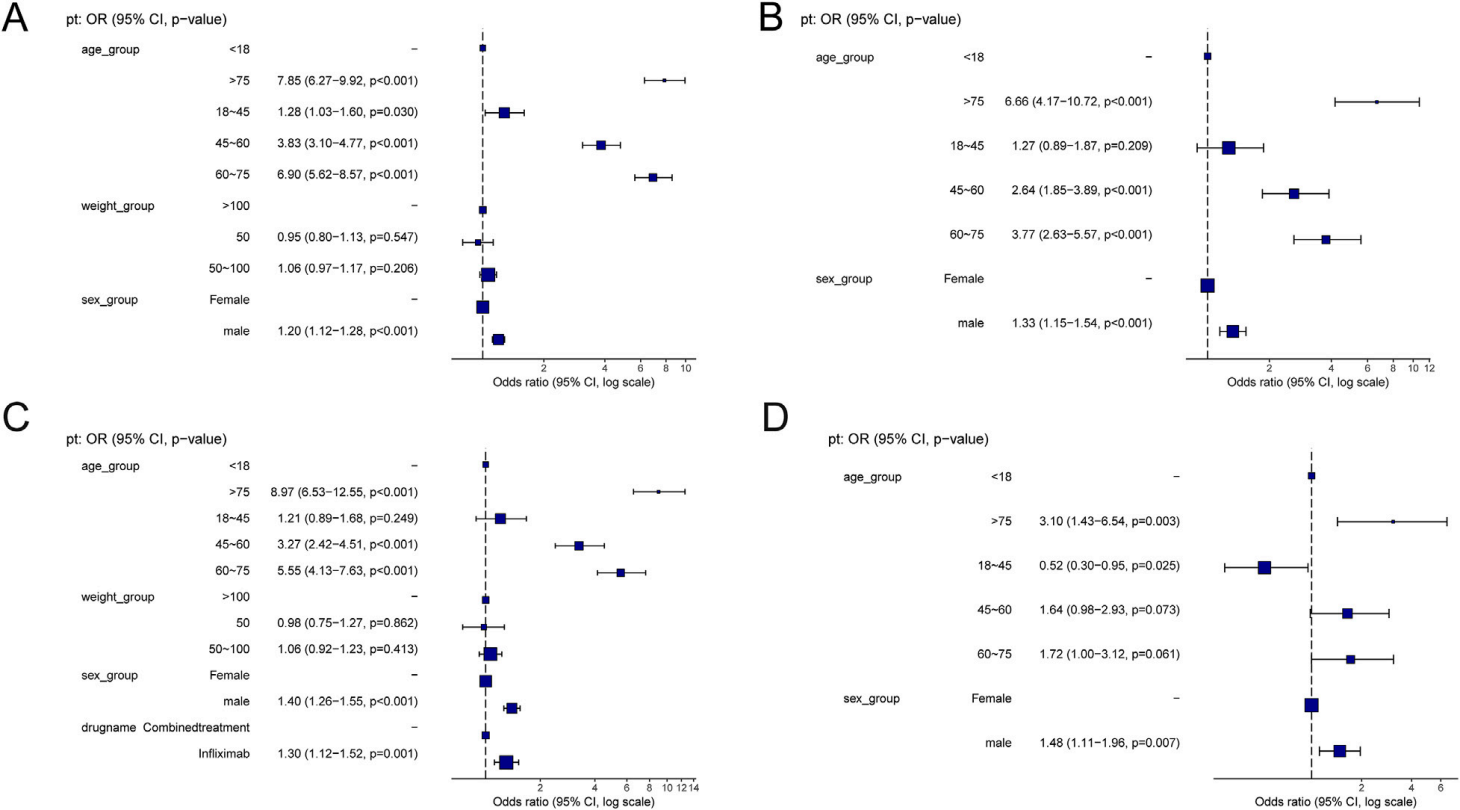
**(A)** Results of Multifactorial Logistic Regression in the Infliximab Group. **(B)** Results of Multifactorial Logistic Regression in the combined treatment Group. **(C)** Multifactorial logistic regression results for the infliximab and combined treatment groups. **(D)** Multifactorial logistic regression results for the azathioprine and combined treatment groups.

Supplementary Table S2 presents a summary of the results from the three WSP trials. The data indicate that the group receiving only infliximab and azathioprine exhibited a random failure-type profile, suggesting that the hazard may be increasing over time. In contrast, the combination group demonstrated an early failure-type profile, which implies that the hazard was decreasing Figure 2 illustrates the number of reported cases of tumor-related adverse events across different treatment groups in each country. In the infliximab-only and combination groups, Canada reported the highest number of cases, with 3,144 and 195 cases, respectively. This was followed by the United States, which reported 2,970 and 10 cases, respectively. In contrast, the azathioprine-only group had the highest number of reported cases in the United States, totaling 41 cases. The findings related to tumor-associated adverse signals are presented in Supplementary Figure S2. A total of 67 significant signals were identified in the infliximab-only group, while 4 significant signals were detected in the azathioprine-only group, and 21 significant signals were observed in the combination group. Figures 3A–C illustrates the top ten relative odds ratios (ROR) and their 95% confidence intervals for the frequency of tumor-associated significant signals across various groups, presented in the format of a forest plot. Figures 3D–F, on the other hand, illustrates the distribution of time to diagnosis across the different groups. The results indicated that in the infliximab-only and azathioprine groups, the time to tumor diagnosis was primarily concentrated between 1 and 5 years, whereas no significant difference was observed in the combination group. The findings from one-way logistic regression analyses revealed that, within the infliximab-only group, there were significant differences in age, body weight, and sex. In contrast, the azathioprine-only group did not demonstrate significant differences in age, sex, or body weight. Furthermore, significant differences in sex and age were also identified in the combination group, as outlined in Supplementary Table S1 For meaningful single-factor results, we further performed multifactor logistic regression analyses. The forest plot presented in Figure 4A, B depicts the outcomes of the multifactorial logistic regression analysis. Within the infliximab-only cohort, adult participants exhibited a heightened risk of developing cancer in comparison to minors. Furthermore, male individuals demonstrated a 1.2-fold increased risk of cancer relative to their female counterparts, while body weight did not reveal any statistically significant differences. In the combination treatment group, middle-aged and older adults aged over 45 years displayed an elevated risk of tumors when compared to minors, with the risk for men being 1.33 times greater than that for women.

### 3.3 Comparative statistical analysis of adverse events in different treatment groups

**FIGURE 5:**
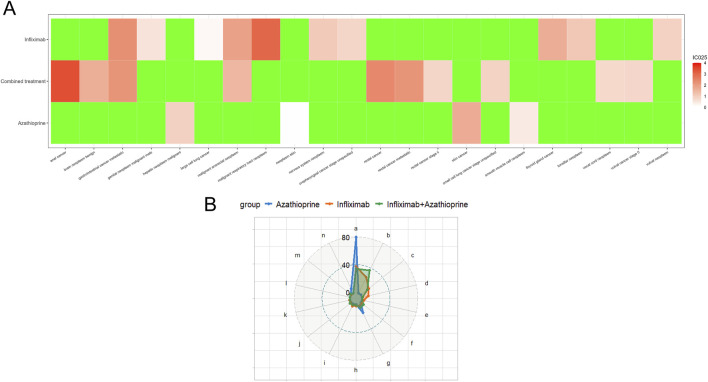
**(A)** Top Ten Risks of Adverse Events Associated with different groups According to the Reported Odds Ratio (ROR). (In this context, ROR refers to the reported odds ratio, IC denotes the information component, and IC025 represents the lower limit of the 95% confidence interval for the reported odds ratio). **(B)** Distribution of SOC Across Various Treatment Groups. (notes: a: Skin and subcutaneous tissue disorders; b: Gastrointestinal disorders; c: Respiratory, thoracic and mediastinal disorders; d: Other neoplasms benign, malignant and unspecified (incl cysts and polyps); e: Renal and urinary disorders; f: Investigations; g: Hepatobiliary disorders; h: Endocrine disorders; i: Reproductive system and breast disorders; j: Nervous system disorders; k: Ear and labyrinth disorders; l: Eye disorders; m: Blood and lymphatic system disorders; n: Musculoskeletal and connective tissue disorders).

**FIGURE 6:**
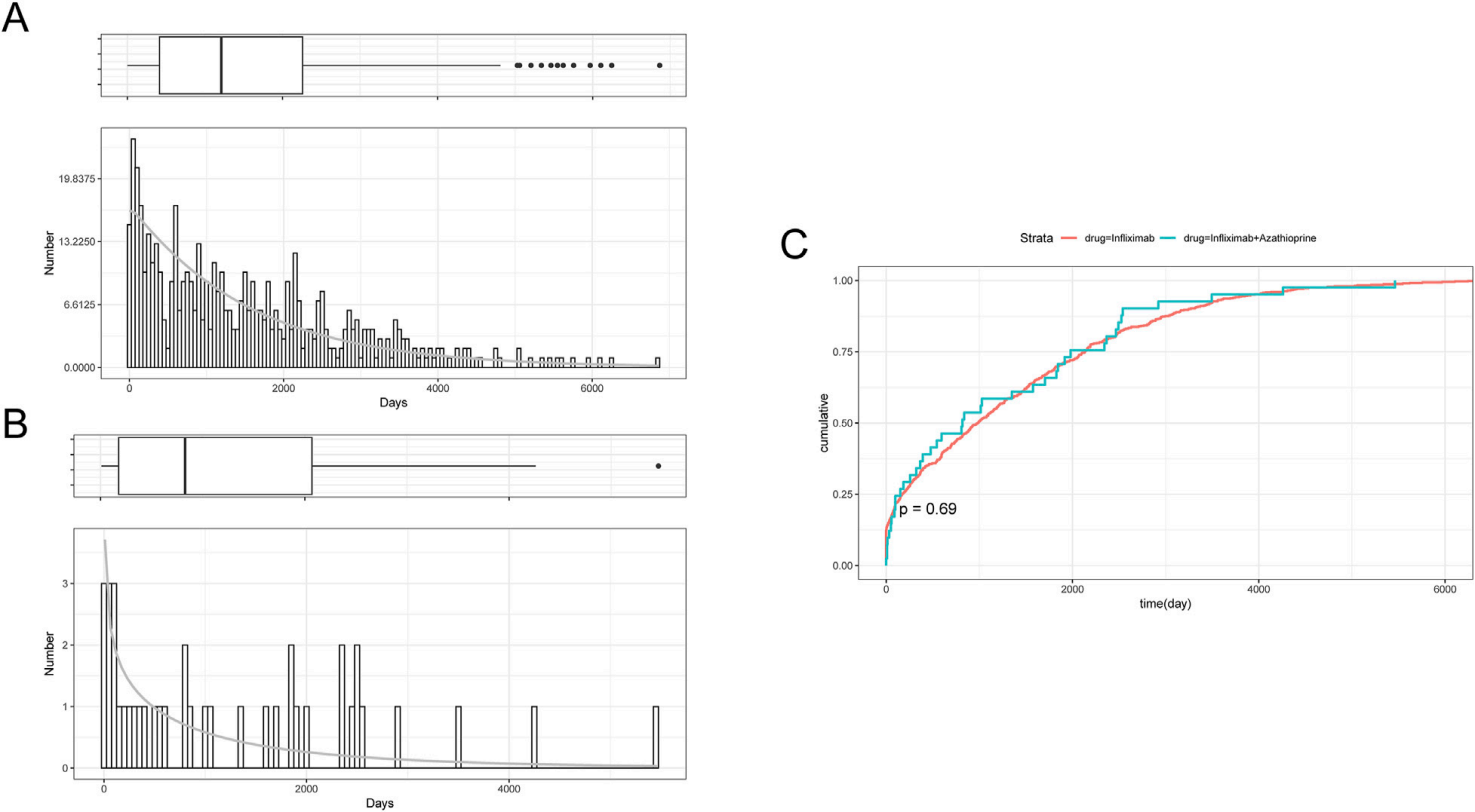
**(A)** Histogram of Time to Onset of Tumor-Associated Significant signals in the Infliximab Group. **(B)** Histogram of Time to Onset of Tumor-Associated Significant signals in the combined treatment Group. **(C)** Cumulative Incidence in the Infliximab and combined treatment Groups.

Table 2 delineates the clinical characteristics associated with the tumor-related significant signals. The findings indicated that females remained the predominant demographic across the various groups, with most weights concentrated within the range of 50–100 kg. In terms of age distribution, patients receiving infliximab alone were primarily in the 60–75 year age group, those on azathioprine were predominantly in the 18–45 year age group, and patients on the combination therapy were mainly in the 45–60 year age group. Figure 5A presents a heatmap depicting the three treatment groups ranked among the top ten based on the Risk of Occurrence Ratio (ROR) for tumor-associated significant signals. The findings indicate that the infliximab group exhibits the highest risk of malignant respiratory tumors, the azathioprine group demonstrates the highest risk of skin cancer, and the combination therapy group is associated with the highest risk of anal cancer. The results presented in the radargram depicted in Figure 5B indicate that disorders of the skin and subcutaneous tissue were the most prevalent across the various treatment groups. Notably, hematological and lymphatic disorders were observed in patients receiving the combination therapy, whereas musculoskeletal and connective tissue disorders were reported in a single case among patients treated exclusively with azathioprine. Supplementary Table S1 presents the findings from the one-way regression analysis comparing the combination therapy to monotherapy. The analysis revealed significant differences in age, weight, sex, and medication between the combination group and the infliximab-only group. Additionally, when comparing the combination therapy to the azathioprine-only group, significant differences were observed in age and sex, while no significant differences were noted in weight and medication. Furthermore, we conducted a multifactorial logistic regression analysis, which indicated that, after controlling for age, sex, and weight, the risk of cancer was 1.3 times greater with infliximab monotherapy compared to the combination therapy (refer to Figure 4C for further details). In the comparative analysis between the combination therapy group and the azathioprine-only group, no statistically significant differences were observed in the risk of tumor development associated with the medication. Furthermore, the incidence of tumors was found to be lower in individuals aged 18 to 45 compared to minors. Additionally, the risk of tumors was 1.48 times greater in males than in females. Figure 6A, B presents histograms illustrating the time to onset for infliximab and combined treatment. The median time to onset was found to be 984.5 days (range: 169–2133.75 days) for the infliximab-only group and 818 days (range: 152–1979 days) for the combination group. Table 3 illustrates the differences in the time to onset of tumor between patients receiving a combination of drugs and those receiving monotherapy. The results indicate that there is no statistically significant difference in the time to onset of tumor between the two groups when compared to other drug treatments. Additionally, Figure 6C presents the cumulative incidence rates for the combination drugs and monotherapy, analyzed using the log-rank test. The analysis further confirms that there is no significant difference in cumulative incidence rates between the combination drugs and monotherapy.

**TABLE 2:** Clinical characteristics of tumor-related significant signals adverse reactions in different groups.

Characteristics	Infliximab (n, %)	Azathioprine (n, %)	Combined treatment (n, %)
Sex			
Female	810 (51.3)	11 (50)	56 (50)
Male	735 (46.55)	8 (36.36)	54 (48.21)
Missing	34 (2.15)	3 (13.64)	2 (1.79)
Weight			
<50 kg	49 (3.1)	0	4 (3.57)
50∼100 kg	832 (52.69)	3 (13.64)	64 (57.14)
>100 kg	119 (7.54)	0	10 (8.93)
Missing	579 (36.67)	19 (86.37)	34 (30.36)
Age			
<18	5 (0.32)	0	2 (1.79)
18∼45	190 (12.03)	7 (31.82)	21 (18.75)
45∼60	371 (23.5)	5 (22.73)	40 (35.71)
60∼75	434 (27.49)	3 (13.64)	17 (15.18)
>75	157 (9.94)	2 (9.09)	5 (4.46)
Missing	422 (26.73)	5 (22.73)	27 (24.11)

**TABLE 3:** Comparison of time to onset of tumor-associated significant signals between combined treatment and Groups A and B.

Group	Case reports	Median (d) (25%–75%)	Z	P
Infliximab	604	984.5 (169–2133.75)	0.176	0.86
Combined treatment	41	818 (125–2,161)		
Azathioprine	5	700 (347–2020)	0.93	0.945
Combined treatment	41	818 (152–1,979)		
Infliximab	604	984.5 (169–2133.75)	0.93	0.926
Azathioprine	5	700 (669–1,729)		

## 4 Discussion

Although numerous studies conducted both domestically and internationally have demonstrated the multiple benefits of combining anti-TNF drugs with immunosuppressants, the question of whether this combination increases the risk of tumorigenesis remains a significant topic of academic debate. Based on data derived from pharmacokinetic studies, a hypothesis has been proposed suggesting that infliximab exhibits greater immunogenicity in comparison to adalimumab. Consequently, the administration of infliximab in conjunction with immunosuppressants may yield additional advantages, particularly with respect to achieving elevated drug concentrations (Gibson et al., 2020). Although methotrexate elevates levels of anti-tumor necrosis factor drugs and diminishes immunogenicity, studies comparing combination therapy involving methotrexate and infliximab to infliximab monotherapy have demonstrated no significant differences in clinical remission rates (Targownik et al., 2020). Consequently, the combination of infliximab and azathioprine has emerged as the most frequently utilized combination therapy in clinical practice. The FAERS, as a global pharmacovigilance database, is capable of swiftly and scientifically evaluating the relationship between pharmaceuticals and adverse reactions. Thus, this study aims to employ the FAERS database for data mining to investigate whether the combination of these two agents elevates the risk of tumor development. This will be achieved by comparing the combination therapy with monotherapy in terms of clinical baseline characteristics, time to onset, and other relevant factors, thereby providing further guidance for clinical medication decisions.

The analysis of reporting duration indicates a consistent year-on-year increase in the incidence of adverse reactions associated with both the sole use of the drug and its combination with other therapies. Notably, the highest frequency of reported adverse reactions for infliximab, whether used alone or in combination, occurred in 2023, while the peak for azathioprine was recorded in 2019. These findings suggest a growing awareness of drug-induced tumor-related adverse reactions among the public. Furthermore, this trend serves as a cautionary signal for the administration of these drugs in other countries, highlighting the necessity for enhanced monitoring and reporting of tumor occurrences linked to these medications. Following the application of four data mining methodologies, our findings indicate that females constituted the predominant demographic experiencing adverse reactions, both with infliximab and azathioprine administered individually, as well as in conjunction with one another. This observation aligns with existing literature that suggests a higher prevalence of autoimmune diseases among females (Ngo et al., 2014). Research has indicated that variations in immune response may be associated with factors such as sex hormones and the phenomenon of X chromosome escape. Estrogen has been shown to enhance immune function by facilitating the activation of antigen-presenting cells, including macrophages and dendritic cells, thereby activating the Toll-like receptor (TLR) signaling pathway and promoting both Th1 and Th2 cell responses. In contrast, androgens tend to suppress immune activity by inhibiting the activation of dendritic cells, the initiation of antigen presentation, and the overall immune response, as well as by restraining the proliferation of Th1 and Th2 cells. To achieve a balance in gene expression between males and females, females employ X chromosome inactivation (XCI), which results in the formation of a transcriptionally silenced inactive X chromosome (Xi) that helps maintain this balance. However, it is important to note that XCI does not completely eliminate all gene expression, leading to the phenomenon known as X chromosome escape. Given that the X chromosome harbors numerous immune-related genes, the escape from XCI may enhance the immune response in females (Xing et al., 2022). The study indicates that X chromosome dosage may significantly contribute to the heightened risk of autoimmune diseases in both humans and mice. Specifically, the Xist-RNP complex, which is exclusively expressed in female individuals, may function as an autoantigenic scaffold that promotes the development of sex-preferred autoimmune diseases (Dou et al., 2024). Furthermore, several genes that are not regulated by hormones and are not situated on the sex chromosomes exhibit sex-biased expression. Nevertheless, males demonstrate a higher susceptibility to malignant tumors and are more vulnerable to severe viral, bacterial, and fungal infections compared to females. This disparity may be attributed to differences in the immune cell responses between males and females (Huang et al., 2021; Jacobsen and Klein, 2021). Our findings further substantiate this phenomenon: in addition to the absence of a sex difference in the azathioprine-only group, the risk of cancer was found to be higher in men compared to women in both the infliximab-only and combination groups. In terms of age, our findings indicate that, except for the azathioprine group, where age did not emerge as an independent risk factor, there is a higher incidence of cancer among middle-aged and elderly individuals compared to minors. This trend may be attributable to a variety of factors. Research indicates that individuals with autoimmune disorders exhibit elevated levels of inflammation within their bodies. This inflammation is associated with accelerated aging, and as the aging process progresses, there is a notable increase in the accumulation of inflammatory factors. Furthermore, chronic inflammation has been shown to facilitate the development, progression, and metastasis of cancer (Bogeska et al., 2022; Huang et al., 2021). In terms of body weight, the three groups predominantly fell within the range of 50–100 kg. While some studies have indicated that obesity may accelerate tumor progression by promoting the release of inflammatory factors (Kolb et al., 2016) and inducing cellular senescence (Fournier et al., 2023), our study did not identify body weight as an independent risk factor. This finding may be attributed to the absence of data regarding the patients’ height and the lack of a scientifically rigorous definition of obesity subgroups. Furthermore, our analysis revealed that among the three drug groups examined, the highest incidence of reported cases was associated with skin-related tumors, followed by gastrointestinal tumors. Additionally, hematological and lymphatic disorders were observed in patients receiving a combination of these drugs. These findings align with prior research indicating that skin cancer is a prevalent malignancy following the administration of TNF-α inhibitors, and that the early use of azathioprine serves as an independent risk factor for malignancies affecting the hematopoietic system (Beaugerie et al., 2009; Kakuta et al., 2022; Khan et al., 2022; Peyrin-Biroulet et al., 2011; Sato et al., 2020).

Previous research has indicated that prolonged use of azathioprine may be associated with an elevated risk of tumorigenesis. The underlying mechanism may involve the structural similarity of 6-thioguanine nucleotides (6-TGN) to guanine, which allows for their incorporation into ribonucleotides. This incorporation can lead to base instability and mismatches, as well as increased susceptibility to mutagenic factors such as ultraviolet radiation and reactive oxygen species. These factors may induce genetic mutations. Furthermore, azathioprine may directly impair the functionality of cytotoxic T cells and natural killer cells, thereby inhibiting cell-mediated immune surveillance. This impairment can facilitate the proliferation of potentially oncogenic lymphocytes infected with Epstein-Barr virus (EBV), human papillomavirus (HPV), and other pathogens, consequently prolonging inflammation and promoting tumor development. The extensive proliferation of lymphocytes may further delay the resolution of inflammation, and the persistent stimulation from chronic inflammation may accelerate the process of tumorigenesis. (Beaugerie, 2012; Kotlyar et al., 2011; Smith et al., 2010). A clinical study has demonstrated that patients undergoing long-term treatment with azathioprine exhibit a minimal risk of developing tumors (Ranjan et al., 2022). In contrast, our study concluded that the combination of infliximab did not elevate the risk of tumorigenesis when compared to monotherapy. Additionally, there is no substantial evidence to suggest a strong association between TNF-α inhibitors and an increased risk of malignancy (Mercer et al., 2015). Our study indicates that for patients receiving infliximab monotherapy, the addition of combination therapy not only does not elevate the risk of tumor development but may actually reduce it. This observation may be attributed to the inherent association between autoimmune diseases and an increased risk of tumorigenesis. Azathioprine appears to diminish the immunogenicity of the patient, which subsequently leads to a reduction in the formation of antibodies against infliximab, a decrease in the inflammatory response, an improvement in the response rate, and an increase in the remission rate (Kang et al., 2018; Luber et al., 2021; Luber et al., 2021).

Over the past 5 decades, significant advancements have been achieved in our comprehension of carcinogenesis. Nevertheless, our grasp of the molecular foundations of systemic manifestations and the root causes of cancer-related mortality remains insufficient. This limitation is attributable to the intricate nature of tumors as a systemic disease, which encompasses various dimensions of tumorigenesis and promotion, the tumor microenvironment and immune macroenvironment, as well as factors such as aging, metabolism, obesity, malignant stroma, circadian rhythms, neurological interactions, tumor-associated thrombosis, and the microbiome (Swanton et al., 2024). Consequently, the analysis of tumor onset timing is essential for facilitating early detection and treatment, which are pivotal for improving patient prognosis and quality of life. To enhance the accuracy of the onset time analysis, we excluded any erroneous or missing data. The Weibull parameters can be employed to predict the duration preceding the onset of adverse reactions, thereby offering valuable insights for the pharmacological management of patients in clinical practice (Mazhar et al., 2021). The findings from the three WSP trials indicate that the group receiving infliximab and azathioprine exhibited a random failure-type profile, suggesting that the estimated hazard of tumor onset occurred consistently over time. This implies that the emergence of tumors associated with the use of the drug alone was random and not significantly influenced by external factors. Conversely, the combination therapy demonstrated an early failure-type profile, characterized by a decreasing hazard over time. This observation underscores the importance of closely monitoring tumor emergence in the initial stages of combination therapy to effectively manage symptoms and mitigate the risk of more severe adverse events. Furthermore, our analysis of the median time to tumor onset and cumulative incidence revealed no statistically significant differences between the combination therapy and monotherapy in terms of median time to tumor onset or cumulative incidence.

Despite the valuable insights obtained from this study, several limitations must be acknowledged. First, the database utilized contained a restricted number of cases, particularly within the azathioprine group, which may have influenced the results and necessitates validation through further research. Second, although we employed four distinct approaches to signal mining of adverse reactions, a causal physiological link could not be established, indicating that their association requires additional assessment and validation. Furthermore, the FAERS database predominantly reflects populations from Europe and North America, with limited representation from Asia and other regions, thereby introducing geographical limitations that may not accurately represent the broader context. Lastly, the FAERS database relies on self-reporting, which may be susceptible to omissions, misreporting, and issues related to data quality. Consequently, it is recommended that a more comprehensive investigation into the risk of adverse events associated with combination drug regimens across various geographic regions be conducted to enhance the assessment of their safety.

## 5 Conclusion

After controlling for variables such as age, sex, and body weight, the combination therapy did not significantly increase the risk of tumor development when compared to the azathioprine-only group. Additionally, among patients receiving infliximab as monotherapy, the combination therapy not only did not elevate the risk of tumor development but also appeared to reduce it.

## Data Availability

The original contributions presented in the study are included in the article/Supplementary Material, further inquiries can be directed to the corresponding authors.
